# Nanoparticle-Mediated Gene Silencing for Sensitization of Lung Cancer to Cisplatin Therapy

**DOI:** 10.3390/molecules25081994

**Published:** 2020-04-24

**Authors:** Daniel P. Feldmann, Joshua Heyza, Christoph M. Zimmermann, Steve M. Patrick, Olivia M. Merkel

**Affiliations:** 1 Department of Oncology, School of Medicine and Barbara Ann Karmanos Institute, Wayne State University, Detroit, MI 48201, USA; dpfeldma@gmail.com (D.P.F.); heyzajo1@msu.edu (J.H.); patricks@karmanos.org (S.M.P.); 2 Department of Pharmaceutical Sciences, School of Pharmacy, Wayne State University, Detroit, MI 48201, USA; 3 Department of Pharmacy, Ludwig-Maximilians-Universität München, 81377 München, Germany; christoph.zimmermann@cup.uni-muenchen.de

**Keywords:** lung cancer, cisplatin resistance, ERCC1, siRNA, nanoparticle, microfluidics

## Abstract

Platinum-based chemotherapy remains a mainstay treatment for the management of advanced non-small cell lung cancer. A key cellular factor that contributes to sensitivity to platinums is the 5′-3′ structure-specific endonuclease excision repair cross-complementation group 1 (ERCC1)/ xeroderma pigmentosum group F (XPF). ERCC1/XPF is critical for the repair of platinum-induced DNA damage and has been the subject of intense research efforts to identify small molecule inhibitors of its nuclease activity for the purpose of enhancing patient response to platinum-based chemotherapy. As an alternative to small molecule inhibitors, small interfering RNA (siRNA) has often been described to be more efficient in interrupting protein–protein interactions. The goal of this study was therefore to determine whether biocompatible nanoparticles consisting of an amphiphilic triblock copolymer (polyethylenimine-polycaprolactone-polyethylene glycol (PEI-PCL-PEG)) and carrying siRNA targeted to ERCC1 and XPF made by microfluidic assembly are capable of efficient gene silencing and able to sensitize lung cancer cells to cisplatin. First, we show that our PEI-PCL-PEG micelleplexes carrying ERCC1 and XPF siRNA efficiently knocked down ERCC1/XPF protein expression to the same extent as the standard siRNA transfection reagent, Lipofectamine. Second, we show that our siRNA-carrying nanoparticles enhanced platinum sensitivity in a p53 wildtype model of non-small cell lung cancer in vitro. Our results suggest that nanoparticle-mediated targeting of ERCC1/XPF is feasible and could represent a novel therapeutic strategy for targeting ERCC1/XPF in vivo.

## 1. Introduction

Lung cancer is the leading cause of cancer related death in the United States with an estimated 228,150 new cases and 142,670 deaths in the Unites States alone in 2019. Lung cancer accounts for 12.9% of all new cancer cases and 23.5% of all cancer-related deaths in the United States [1]. In addition, lung cancer is the most common cancer worldwide and the leading cause of cancer death in both men and women. Due to a lack of pronounced symptoms, the majority of lung cancer cases are diagnosed at late stage disease. More specifically, patients who are diagnosed with localized disease have a 5-year survival rate of 57% while those diagnosed with late stage disease have a 5-year survival rate of 5.2% [1]. Lung cancer is categorized into two main types with the majority of cases being classified as either small cell lung cancer (SCLC) or non-small cell lung cancer (NSCLC) [2]. Non-small cell lung cancer is the most common type of lung cancer and accounts for approximately 85%–90% of all diagnosed lung cancers. When localized, resection of the primary lung tumor offers the best therapeutic option for NSCLC. For metastatic NSCLC, treatment usually contains a platinum agent in combination with immunotherapy unless a tumor-specific, targetable mutation is detected (e.g., ALK or EGFR mutations). Platinum analogues have remained a mainstay for the treatment of advanced NSCLC for almost 50 years, however resistance to these agents commonly occurs. Thus, identifying new targets for improving platinum response in lung cancer is of substantial need and clinical importance. In addition to platinum analogues, the FDA approval of anti-PD-L1 therapy (given as single agent in ~15% of cases or with a platinum in ~85% of NSCLC cases) for first-line treatment of NSCLC has led to substantial clinical benefits for patients [3]. However, resistance to these agents remains a major limitation in achieving cures. 

Platinum analogues elicit their anti-tumor effects by directly binding to guanines in the DNA leading to growth arrest and apoptosis (reviewed in [4]). Similar to other DNA alkylating agents such as nitrogen mustards and mitomycin C, platinums are multifunctional drugs that cause a variety of DNA lesions, including DNA intrastrand crosslinks and interstrand crosslinks. The resulting lesions are highly distorting to the normal structure of the DNA helix and require a variety of DNA repair pathways to effectuate repair, including Nucleotide Excision Repair (NER), Interstrand Crosslink Repair (ICL-R) and Homologous Recombination (HR). One DNA repair protein that has been implicated in the resistance of NSCLC following cisplatin treatment is the excision repair cross-complementation group 1 (ERCC1) protein. Together with ERCC4 (xeroderma pigmentosum group F, XPF), ERCC1 forms a structure-specific endonuclease which plays a rate-limiting role in the nucleotide excision (NER) pathway that recognizes and removes DNA adducts that are formed during cisplatin treatment. ERCC1/XPF plays critical roles in NER for the removal of platinum-DNA intrastrand crosslinks and critical roles in ICL-R for removal of interstrand crosslinks and is thought to be indispensable for platinum-DNA adduct repair. Along these lines, we have previously shown that downregulation of ERCC1/XPF enhances platinum response in vitro in lung and ovarian cancer cell lines [5]. In addition, many in vitro and clinical studies have linked resistance to platinum compounds to the levels of ERCC1 mRNA and protein in NSCLC where up to ~40% of tumors harbor low to undetectable ERCC1 expression [6,7,8,9]. The relationship between ERCC1 expression levels and cisplatin sensitivity has been corroborated by several retrospective clinical studies that report altered expression levels of ERCC1 in patients with NSCLC and other cancers suggesting that ERCC1/XPF could be a potential therapeutic target to enhance platinum response in several tumor types [7,10,11,12,13,14,15]. Additionally, a clinical study involving 761 NSCLC tumors concluded that a significant benefit was associated with the absence of ERCC1, and prolonged survival was observed among patients with low ERCC1 that received cisplatin therapy [8]. Finally, we recently reported that effective use of ERCC1 as a biomarker for predicting lung cancer response to platinum-based chemotherapy depends on the presence of wildtype p53, suggesting that the biological consequences of loss of ERCC1 in tumors may be more complex than previously thought [16]. 

Considering the evidence that ERCC1 could be a potential therapeutic target for enhancing responses to platinum-based chemotherapy in lung cancer, the development of small molecule inhibitors of XPF nuclease activity has been the focus of several studies from multiple research groups [17,18,19,20,21,22]. We previously identified NSC16168 as a potent inhibitor of ERCC1/XPF activity capable of enhancing platinum response both in vitro and in vivo [18]. In addition, we identified epigallocatechin catechin gallate (EGCG) and its pro-drug, Pro-EGCG, as an ERCC1/XPF inhibitor capable of enhancing platinum response in vivo [17]. Several other groups have also focused on the development of novel and improved ERCC1/XPF inhibitors [19,20,21]. While these small-molecule approaches to targeting ERCC1/XPF undergo further preclinical development, here we present evidence for successful targeting of ERCC1/XPF by small interfering RNA (siRNA)-containing micelleplexes in vitro which can enhance platinum response in clonogenic assays. 

One possible and underexplored strategy to downregulate elevated levels of ERCC1 would involve nucleic acid-based therapy. Therapeutic small interfering RNA (siRNA) containing a specific sequence targeted toward downregulating a certain gene can be synthesized and applied in mammalian cells to efficiently degrade targeted mRNA. Therefore, administration of siRNA in vivo to silence disease related genes has often been described to be a safe and potent therapeutic approach [23,24]. With the FDA approval of now two siRNA-based drugs, RNA interference therapy has become a reality for rare diseases with pathology located in the liver [25,26]. However, in vivo targeted delivery of siRNA to cells and tissues outside the liver remains challenging. Considering the large size, instability and negative surface charge, siRNA is easily enzymatically degraded, aggregates with serum proteins and is unable to cross physiological membranes readily [27]. To overcome these challenges, two categories of siRNA delivery vehicles have been explored, viral and non-viral vectors. Due to the safety issues of viral vectors (e.g., immunogenesis, insertional mutagenesis and non-specific transgene delivery), non-viral delivery vectors are a more attractive and favorable approach to delivering siRNA. Polymeric delivery systems, a major class of non-viral vectors, attract great research attention, because of their flexible and modifiable chemical structure. Polymeric delivery systems have the potential to overcome both extracellular biological barriers (e.g., stability in blood stream, crossing biological membranes) and intracellular biological barriers (e.g., endosomal escape, cytoplasmic siRNA release), all while maintaining the high capacity of siRNA loading, biocompatibility and the ability to co-deliver small molecular drugs [28,29,30,31]. 

In this study, the amphiphilic triblock copolymer consisting of polyethylenimine-polycaprolactone-polyethylene glycol (PEI-PCL-PEG; short PPP) was used as one such polymeric delivery system. Polyethylenimine (PEI) is one of the most widely studied non-viral vectors of siRNA due to its high transfection efficiency and modifiable chemical structure [32,33]. Although PEI has been reported to be an efficient carrier and transfection reagent, homologues with larger molecular weight and a higher degree of branching are often required to achieve therapeutically relevant levels of transfection [34]. Conversely, this higher molecular weight also elicits stronger toxicity towards cells which necessitates the use of modification to increase biocompatibility [35]. One such modification involves the use of polyethylene glycol (PEG) that not only reduces the polymer’s toxicity, but also increases its circulation profile, helps to avoid macrophage detection and decreases the interaction with serum proteins [36]. Finally, the hydrophobic polymer polycaprolactone (PCL) is used to drive micelle formation and to aid in polymer cleavage due to its susceptibility toward hydrolytic degradation [37]. Previous studies performed with PEI-PCL-PEG micelleplexes demonstrated their ability to efficiently deliver siRNA in vitro to mediate sustained protein knockdown and display long-circulation profiles in vivo [38,39,40,41]. Here, as a proof of principle we hypothesized that by effectively preparing reproducible and monodisperse nanoparticles containing siRNA through microfluidics and by delivering siRNA inside triblock copolymer PEI-PCL-PEG nanoparticles, we could sensitize NSCLC cells toward cisplatin therapy.

## 2. Results

### 2.1. In Vitro ERCC1-XPF Protein Knockdown 

**Figure 1:**
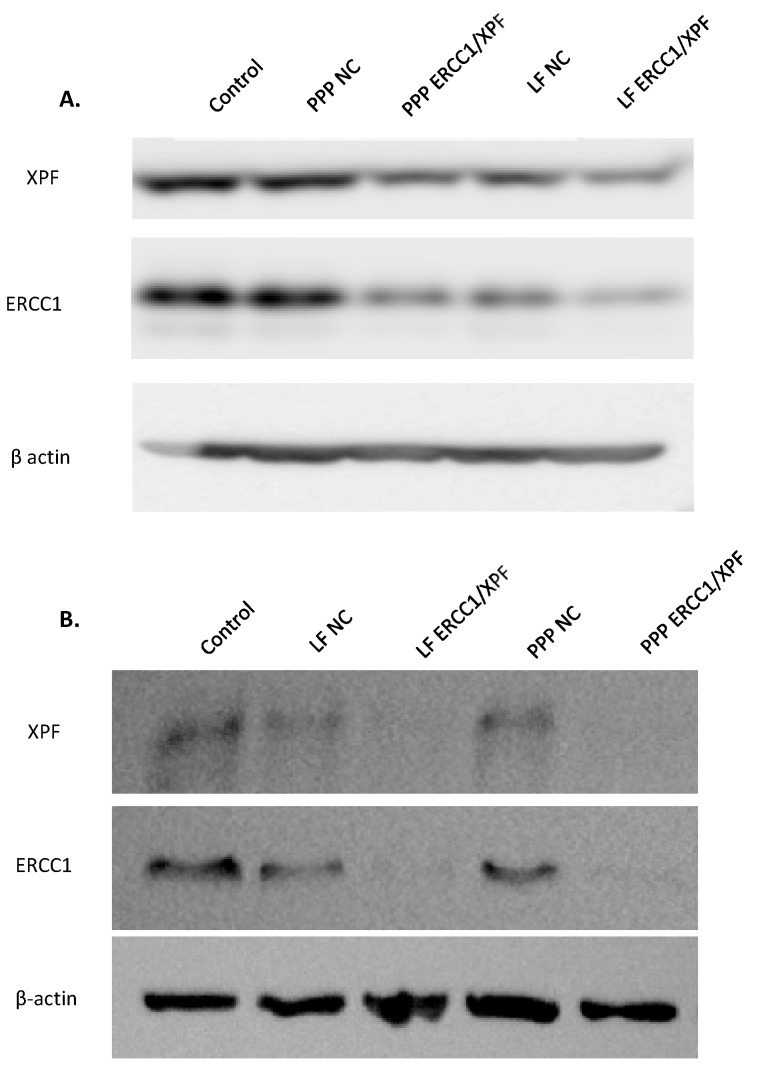
Western Blot analysis of excision repair cross-complementation group 1 (ERCC1) and xeroderma pigmentosum group F (XPF) protein levels within A549 cells following (**A**) single transfection of micelleplexes (PPP) loaded with 50 pmol small interfering RNA (siRNA) after 72 h and (**B**) double transfection of micelleplexes (PPP) loaded with 100 pmol siRNA after 72h. Lipofectamine 2000 (LF) lipoplexes were prepared according to manufacturer’s protocol with 50 pmol (A) and 100 pmol (B) siRNA (n = 2).

With the previous optimization of micelleplex microfluidic mixing and in vitro/in vivo proof-of-concept of siRNA delivery with <200 nm nanoparticles in our past study, we set out to optimize the knockdown of the proteins ERCC1 and XPF [38]. For preliminary experiments, A549 lung cancer cells were transfected with micelleplexes and Lipofectamine (LF) that contained 50 pmol of ERCC1-XPF siRNA, and protein knockdown was determined after 72 h via Western blot analysis with β-actin as a loading control. Furthermore, negative control (NC) siRNA formulations with PEI-PCL-PEG and LF were included as well. Figure 1A shows that single transfection with 50 pmol of siRNA mediates modest protein knockdown of ERCC1 and XPF for both LF and the micelleplexes after 72 h (for quantification see Figure S1, Supplementary Material). Given these results, we then increased the amount of ERCC1-XPF siRNA that was transfected to 100 pmol and doubled the number of transfections administered over 72 h. Figure 1B shows that with this optimization, significant protein knockdown of ERCC1 and XPF was achieved for both LF and the micelleplexes after 72 h. Quantification of the protein levels (see Figure S2, Supplementary Material) indicates that the PEI-PCL-PEG micelleplexes effectuated ~90% knockdown of ERCC1 and XPF while LF achieved ~90% knockdown of ERCC1 and 72% knockdown of XPF.

### 2.2. In Vitro ERCC1-XPF Gene Knockdown

**Figure 2:**
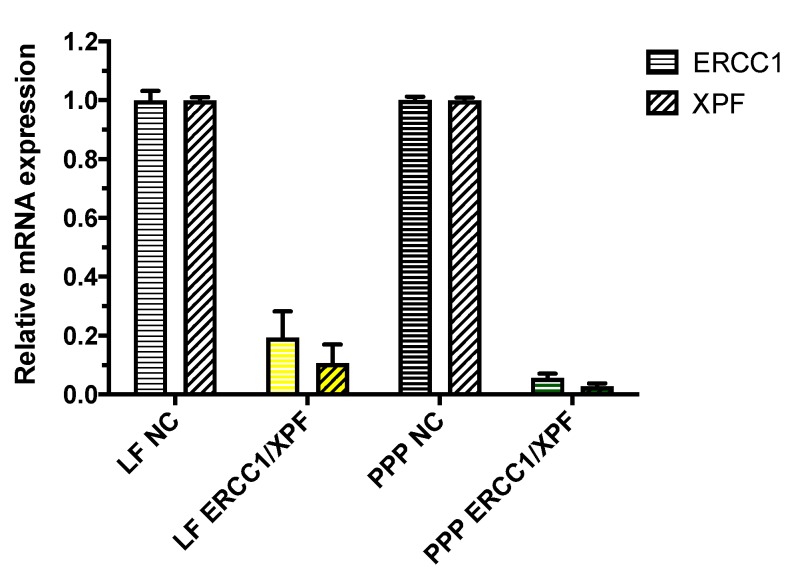
ERCC1 and XPF gene knockdown efficiency was validated in lung adenocarcinoma cells (A549) after 72 h following double transfection of Lipofectamine 2000 (LF) or micelleplexes (PPP) with 100 pmol ERCC1-XPF siRNA or negative control (NC) siRNA. ERCC1 and XPF expression was normalized with GAPDH expression and quantified by real time PCR. Data points indicate mean ± SD. (n = 6).

After optimizing the protein knockdown of ERCC1-XPF, the changes in protein expression following transfection of A549 cells with ERCC1-XPF siRNA were correlated with the changes in transcript level following quantification with real time PCR (qRT-PCR) normalized to GAPDH gene expression. Hence, A549 cells were transfected with PEI-PCL-PEG (PPP) micelleplexes that contained 100 pmol ERCC1-XPF or scrambled siRNA for 72 h. Negative controls consisted of cells transfected with the respective transfection reagent, i.e., LF or PPP micelles, and scrambled control (NC) siRNA, while positive control cells were transfected with siRNA against the respective mRNA, i.e., ERCC1 or XPF, and LF. As shown in Figure 2, A549 cells that were treated with PEI-PCL-PEG micelleplexes showed a significant decrease of both ERCC1 and XPF mRNA levels (>90%) when compared to those cells treated with scrambled siRNA (NC) micelleplexes. Furthermore, ERCC1-XPF mRNA levels in these samples were slightly lower in comparison to cells that were treated with LF.

### 2.3. Colony Survival Assay 

**Figure 3:**
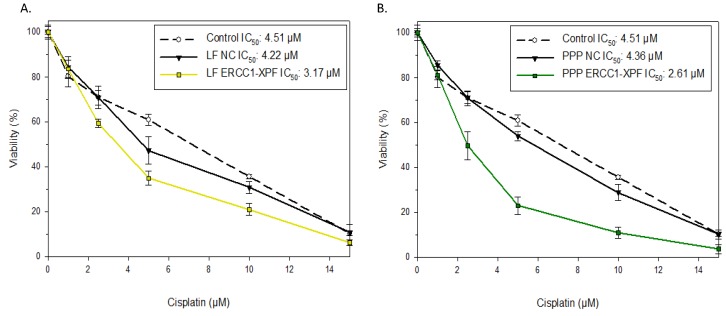
Colony survival assay in A549 cells following transfection with Lipofectamine 2000 (**A**) or PEI-PCL-PEG (PPP) micelleplexes (**B**). Untreated (open circle), negative control (filled triangles) or ERCC1-XPF (filled squares) siRNA transfected cells were treated with increasing doses of cisplatin for 2 h, and cell viability was determined by a clonogenic assay. Results are represented as mean ± SD. IC_50_ values were calculated using Compusyn software.

To assess the effect of ERCC1-XPF knockdown on cell clonogenicity in response to cisplatin, clonogenic assays were performed with untreated, control siRNA-transfected A549 cells, and cells undergone ERCC1-XPF knockdown using either LF or PEI-PCL-PEG micelleplexes to deliver siRNAs. Figure 3A demonstrates a 1.4-fold change in A549 IC_50_ value following ERCC1-XPF knockdown mediated by Lipofectamine transfection and corroborates previous results generated with commercial transfection reagents [5]. Figure 3B shows that ERCC1-XPF knockdown mediated by PEI-PCL-PEG micelleplexes resulted in a 1.6-fold change in IC_50_ when compared to untreated and scrambled sequence siRNA-treated cells (for statistic analysis see Figure S3, Supplementary Material). The slightly higher fold change in IC_50_ values observed in cells treated with micelleplexes compared to those treated with LF may be a result of the higher level of protein and mRNA knockdown presented above.

## 3. Discussion

One of the major mechanisms of action behind cisplatin cytotoxicity is the formation of platinum-DNA adducts that lead to the inhibition of DNA replication and ultimately cell death. Once formed, these adducts are thought to be repaired by multiple DNA repair pathways including, NER, HR, and ICL-R. As such, increased expression of genes in these pathways, including ERCC1/XPF contribute to resistance to platinum-based chemotherapy. One consequence of increased ERCC1/XPF expression is a narrowed therapeutic window for platinum efficacy where the amount of platinum required to inhibit tumor growth leads to dose-limiting toxicities. As such, targeting a well-established DNA repair factor involved in platinum-resistance could be capable of enhancing the therapeutic efficacy of platinum drugs in NSCLC. Given the major role that NER plays in the mechanism of cisplatin induced DNA repair, targeting key components in the pathway presents a novel approach to enhance cisplatin efficacy for cancer treatment. To date, introductory studies involving the use of small molecule inhibitors of the ERCC1-XPF complex have shown the potential to increase cisplatin toxicity in vitro and in vivo but require further pharmacological testing and development in order to generate second generation compounds with maximal inhibitory activity against ERCC1/XPF endonuclease activity [17,18]. Furthermore, the use of RNA interference as a means of inhibiting the ERCC1-XPF complex is an attractive approach and was confirmed in multiple studies to demonstrate effectiveness in increasing cisplatin efficacy within multiple cancer cell lines [5]. However, the use of commercially available lipid based transfection agents to deliver the siRNA used in these studies limits the translation of these treatments into preclinical animal models due to the well documented toxicity profiles associated with their use [5]. Therefore, an alternative delivery vehicle with higher biocompatibility as well as industrially scalable approaches such as microfluidics are desirable to further investigate the use of RNA interference as a means of increasing cisplatin effectiveness in the treatment of cancer. In our study, we utilized the amphiphilic triblock copolymer consisting of polyethylenimine-polycaprolactone-polyethylene glycol (PEI-PCL-PEG) to form micelleplexes with siRNA targeted at ERCC1-XPF.

Initially, we optimized the transfection parameters that would result in the most efficient knockdown of ERCC1 and XPF protein within A549 cells. After a single 72 h transfection of the cells with Lipofectamine 2000 or PEI-PCL-PEG micelleplexes loaded with 50 pmol siRNA, modest protein knockdown was observed by Western blot analysis (Figure 1A). Previous studies have reported on the reduced presence of ERCC1-XPF leading to only mild impairment of NER and suggest the necessity of a near absence of the complex in order to provide the most therapeutic benefit [5]. Therefore, the initial decrease in protein expression following this single transfection was considered insufficient for subsequent therapeutic effects and therefore necessitated the increase of the siRNA dose to 100 pmol along with the change to a double transfection over 72 h. Once these parameters were modified, reduced protein expression following transfection was vastly improved, and Western blot analysis confirmed >90% knockdown of ERCC1 and XPF in those cells treated with the micelleplexes (Figure 1B). The reduced expression of ERCC1 and XPF protein was further complemented by analysis of their transcript levels which showed similar reduction of 90% following the double transfection of micelleplexes over 72 h (Figure 2). 

Finally, we investigated the capacity of the A549 cells to survive and resist the effects of cisplatin treatment following transfection with ERCC1-XPF targeted siRNA. Given the major role that ERCC1/XPF plays in repairing cisplatin-DNA adducts, the reduced expression of the protein complex was expected to lead to increased cell death and reduction in clonogenicity after cisplatin treatment. Results from our clonogenic assay support this hypothesis and demonstrate a 1.6-fold change in the IC_50_ value to cisplatin of those cells that were treated with micelleplexes loaded with targeted siRNA (Figure 3). Additionally, impact on mitochondrial activity was measured in MTT assays (see Figure S4, Supplementary Material). As discussed in other work, forming nanoparticles by microfluidics leads to an increased control over the production process which in turn generates decreased hydrodynamic diameters and narrow dispersity [38,42,43]. Loy et al. produced well-defined and reproducible polyplexes with controlled surface characteristics with the help of microfluidic a self-assembly based on electrostatic interaction [44]. Therefore, microfluidic manufacturing also provides a promising tool for the controlled formation of micelleplexes as it enables a rapid and tunable mixing of both fluids by inducing chaotic advection of the fluid streams and decreasing the average diffusion distance to homogenize the unmixed polymer and siRNA solutions [42]. The resulting nanocarriers are hence expected to mediate highly efficient cellular internalization [45]. Additionally, PEI-PCL-PEG micelleplexes have been shown to deliver siRNA to the cytoplasm without extensive endosomal entrapment [38]. Therefore, combining siRNA delivery with microfluidic PEI-PCL-PEG micelleplexes is a promising approach to enhancing gene silencing in general, and for example, the specific effectiveness of chemotherapy, as shown here. Nonetheless, future optimizations of micelleplex-mediated ERCC1-XPF knockdown—such as increased time periods for the protein knockdown to occur before treating with cisplatin—are required in order to further increase IC_50_ fold change in vitro before beginning in vivo investigations. However, data presented here suggests that targeting of ERCC1/XPF with siRNA containing micelleplexes is both feasible and capable of effectuating platinum sensitivity in a p53 wildtype model of NSCLC in vitro.

## 4. Materials and Methods 

### 4.1. Materials

Hyperbranched polyethylenimine (PEI, 25k Da) was obtained from BASF (Ludwigshafen, DE). Dulbecco’s Modified Eagle’s Medium (DMEM) was purchased from Gibco (Carlsbad, CA, USA). Dulbecco’s phosphate buffered saline (PBS), heat-inactivated fetal bovine serum (FBS), D-(+)-glucose, sodium bicarbonate, sodium pyruvate, 2-mercaptoethanol, dimethyl sulfoxide (DMSO, ≥ 99.7%), Cisplatin (cis-diamminedichloroplatinum(II)), ethylenediaminetetraacetic acid (EDTA, 99.4−100.06%) and trypan blue (0.4%, sterile filtered) were purchased from Sigma-Aldrich (St. Louis, MO, USA). SYBR Gold dye and Lipofectamine 2000 were obtained from Life Technologies (Carlsbad, CA, USA). A 1 mM stock of cisplatin was prepared fresh daily in PBS by vortexing vigorously until the drug dissolved completely. Dicer substrate double-stranded scrambled nonspecific control siRNA was purchased from Integrated DNA Technologies (IDT, Coralville, IA, USA). ON-TARGETplus SMARTpool siRNA targeting ERCC1 and XPF was purchased from Dharmacon (Lafayette, CO, USA).

### 4.2. Synthesis of Triblock Copolymer and Characterization 

Briefly, the triblock copolymer consisting of polyethylenimine-graft-polycaprolactone-block-polyethylene glycol (PEI25k-g-PCL1k-b-PEG5k, short PEI-g-PCL-b-PEG or PPP) was synthesized by coupling the heterobifunctional diblock copolymer acrylated-PCL-b-PEG alkyne to hy-PEI (25 kDa), as previously described [41]. All compounds synthesized were characterized by ^1^HNMR and UV Spectroscopy [39].

### 4.3. Microfluidic Preparation of PEI-PCL-PEG Micelleplexes 

The triblock copolymer was dissolved in RNase free water to yield a 0.5 mg/mL solution and was then filtered through a 0.22 μm filter for sterilization. This stock solution was then diluted to pre-calculated concentrations with a sterile 5% glucose solution to prepare micelleplexes at an N/P (the molar ratio of nitrogen in PEI to phosphate in siRNA) ratio of 6 as described before [38]. To form micelleplexes via a microfluidic mixing chip, 200 μL (minimum volume) of both the diluted PEI-g-PCL-b-PEG and siRNA solution (2 pmol/µL) was transferred into two separate 3 mL sterile syringes (Becton, Dickinson and Company, Franklin Lakes, NJ, USA). These fluid-filled syringes were then loaded onto a KD Scientific 220 syringe pump (KD Scientific Inc., Holliston, MA, USA) and connected to a hydrophobic Dolomite micromixer chip (Dolomite Microfluidics, Charlestown, MA, USA) via 0.25 mm i.d. FEP tubing. Unless otherwise stated, the polymer and siRNA solution were then mixed at a constant flow rate of 0.5 mL/min and the formed micelleplexes were collected after the mixed solution reached steady state flow. Micelleplexes that were prepared via the micromixer chip did not require further incubation at room temperature and were used directly after collection, as described in our previous study [38]. Resulting micelleplexes were previously characterized regarding hydrodynamic diameters, zeta potentials, encapsulation efficiency, siRNA release and uptake mechanisms in lung cancer cells as well as in the lungs of mice [38]. 

### 4.4. Cell Culture

A549 cells are a human adenocarcinoma alveolar-based lung cancer cell line and were obtained from ATCC (LG Promochem, Wesel, Germany). A549 lung adenocarcinoma cells were cultured in DMEM cell culture medium and supplemented with 10% heat inactivated fetal bovine serum, 2.5% GlutaMAX (Gibco) and 1% penicillin/streptomycin (Corning Incorporated, Corning, NY, USA). All cells were maintained in a humidified atmosphere within 75 cm^2^ cell culture flasks (Thermo Fisher Scientific) and 5% CO_2_ at 37 °C.

### 4.5. In Vitro ERCC1-XPF Protein Knockdown and Western Blot Analysis

For validation of protein knockdown, 150,000 A549 cells were seeded and incubated overnight at 37 °C and 5% CO_2_ in a 6-well plate. Treatment groups consisted of untreated/untransfected cells and cells transfected with Lipofectamine 2000 (Life Technologies) lipoplexes containing either scrambled siRNA or siRNA targeted to ERCC1 and XPF, which were prepared according to the manufacturer’s protocol. Briefly, every 10 pmol siRNA were formulated with 0.5 µL Lipofectamine (LF) solution. PEI-PCL-PEG micelleplexes were mixed and cells were transfected as previously described [38]. Cells were then harvested 24 h after the second micelleplex transfection, washed with PBS and lysed in lysis buffer (10 mM Tris pH 8.0, 120 mM NaCl, 0.5% NP-40, 1mM EDTA) containing protease inhibitors (0.5 M PMSF, 1mg/mL leupeptin and 1 mg/mL pepstatin A). Protein was separated on 10% SDS-polyacrylamide gel and transferred onto Immobilon-P PVDF transfer membrane (Millipore). After blocking (5% non-fat dry milk in TBS-Tween), the membranes were probed with primary antibodies recognizing human ERCC1 and XPF with β-actin as loading control. The dilutions of antibodies used were as follows: anti-ERCC1 1:1,000 (Abcam ab76236), anti-XPF 1:1,000 (Santa Cruz sc-136153), anti-β-actin 1: 100,000 (Sigma-Aldrich A5441). The membranes were incubated at RT in blocking buffer with appropriate secondary antibodies at 1:2,000 dilutions and the signal was detected by using an Enhanced Chemiluminescence detection system. Densitometry was conducted using the Bio-Rad Image Lab™ software suite.

### 4.6. In Vitro ERCC1-XPF Gene Knockdown

For gene silencing experiments, 150,000 A549 cells were seeded and incubated overnight at 37 °C and 5% CO_2_ in a 6-well plate. Micelleplexes were freshly prepared via microfluidic mixing with ERCC1 and XPF siRNA or scrambled siRNA at an N/P ratio of 6. Negative controls consisted of untreated cells or cells transfected with control scrambled siRNA while positive control cells were transfected with Lipofectamine 2000 (Life Technologies) lipoplexes, which were prepared as described above. Unless otherwise stated, cells were transfected twice at 24 h intervals in 37 °C and 5% CO_2_ with 200 μL of micelleplex suspension containing 100 pmol siRNA within a total volume of 2 mL of serum-containing cell culture media. Following the second transfection, the culture media was replaced and cells were incubated for 24 h. Cells were then washed with PBS, harvested and processed to isolate total RNA using the PureLink^TM^ RNA mini kit (Life Technologies) according to the manufacturer’s protocol with DNase I digestion (Thermo Scientific). Synthesis of cDNA from total RNA was performed with High Capacity cDNA reverse transcriptase kit (Applied Biosystems, Foster City, USA) as per the manufacturer’s protocol. The primers that amplify ERCC1, XPF and GAPDH (control) were obtained from Integrated DNA Technologies. ERCC1 forward primer: 5′-CTGGAGCCCCGAGGAAGC-3′; reverse primer 5′-CACTGGGGGTTTCCTTGG-3′. XPF forward primer 5′-AGTGCATCTCCATGTCCCGCTACTA-3′; reverse primer 5′-CGATGTTCTTAACGTGGTGCATCAA-3′. GAPDH forward primer 5’-CGC TCT CTG CTC CTC CTG TT-3’; reverse primer 5’-CCA TGG TGT CTG AGC GAT GT-3’. The transcript levels were quantified using PowerUp™ SYBR™ Green master mix (Life Technologies) in a StepOne real-time PCR system using GAPDH as an endogenous control. The percent transcript knockdown was determined from 2^-ΔΔCT^ values.

### 4.7. Colony Survival Assay

A549 cells were seeded at 150,000 cells per well in 6-well plates and incubated overnight at 37 °C and 5% CO_2_. Negative controls consisted of untransfected cells while positive control cells were transfected with Lipofectamine 2000 (Life Technologies) lipoplexes, which were prepared as discussed above. Micelleplexes were made as described previously with 100 pmol ERCC1 and XPF targeted or scrambled siRNA. Following two 24-h transfections, cells were split and seeded at a density of 450–550 cells in 60 mm dishes in complete medium and incubated overnight. The next day, cells were treated with increasing concentrations of cisplatin for 2 h in serum-free cell culture media after which fresh complete medium with antibiotics was exchanged and cells were then allowed to grow for 7 days or until colonies had >50 cells. Finally, plates were rinsed with PBS and colonies were fixed/stained with 0.2% crystal violet prepared in 20% ethanol. Colonies with ≥50 cells were counted using a light microscope. Cell survival was expressed as the ratio of the average number of colonies in drug treated cells versus control cells × 100. The experiment was done in triplicate for each drug concentration. IC_50_ values were calculated using the Compusyn software (ComboSyn Inc, Paramus, NJ, USA).

### 4.8. Statistics

All results are given as mean value ± standard deviation (SD). One-way ANOVA with Bonferroni post hoc post-test, two-way ANOVA and calculation of area under the curve (AUC) were performed in GraphPad Prism software (Graph Pad Software, La Jolla, CA, USA).

## 5. Conclusions

This study successfully demonstrates the ability of PEI-PCL-PEG micelleplexes to efficiently knockdown ERCC1-XPF mRNA and protein expression and enhance cisplatin response and lays the groundwork for future studies to be conducted utilizing this amphiphilic triblock copolymer for siRNA delivery in combination with microfluidic mixing.

## Supplementary Materials

The following are available online, Figure S1: Densitometric analysis of Western Blot after single transfection. Figure S2: Densitometric analysis of Western Blot after double transfection. Figure S3: Statistic evaluation of IC_50_ values from colony survival assays, Figure S4: MTT assays in A549 cells.
