# Supplementary material for: Nanoparticle-Mediated Gene Silencing for Sensitization of Lung Cancer to Cisplatin Therapy
Source: Molecules. 2020 Apr 24;25(8):1994. doi: 10.3390/molecules25081994 (PMC7221615; doi:10.3390/molecules25081994)
Supplement: Supplementary file 1 [file molecules-25-01994-s001.pdf]

molecules

Supplementary Material

Nanoparticle Mediated Gene Silencing for Sensitization of Lung Cancer to Cisplatin Therapy

Daniel P. Feldmann [1,2], Joshua Heyza [1], Christoph Zimmermann [3], Steve M. Patrick [1] and Olivia M. Merkel [1,2,3*]

[1] Department of Oncology, Wayne State University School of Medicine and Barbara Ann Karmanos Institute, Detroit, MI 48201, USA
[2] Department of Pharmaceutical Sciences, Wayne State University, School of Pharmacy, Detroit, MI 48201, USA
[3] Department of Pharmacy, Ludwig-Maximilians-Universität München, 81377 München, Germany
* Correspondence: Olivia.merkel@lmu.de

Received: 14 February 2020; Accepted: 17 April 2020; Published: 24 April 2020

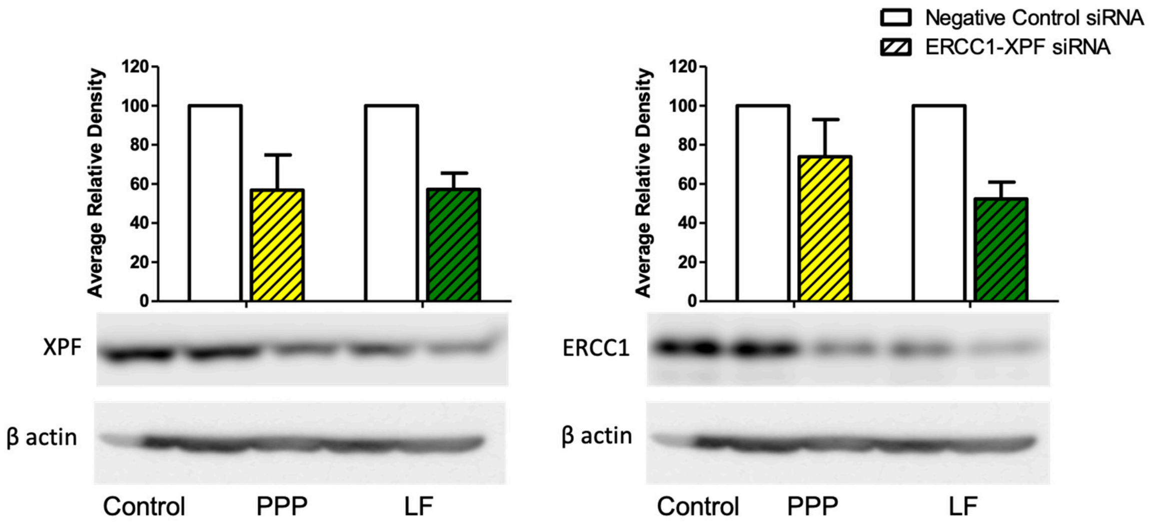

Figure S1. Densitometric analysis of Western Blot after single transfection (compare Figure 1A).

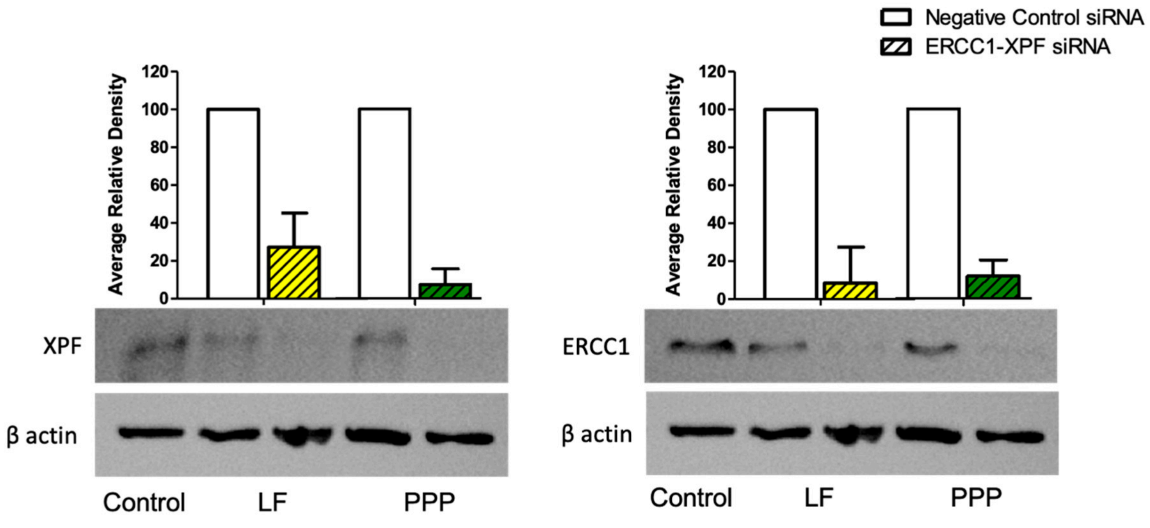

Figure S2. Densitometric analysis of Western Blot after double transfection (compare Figure 1B).

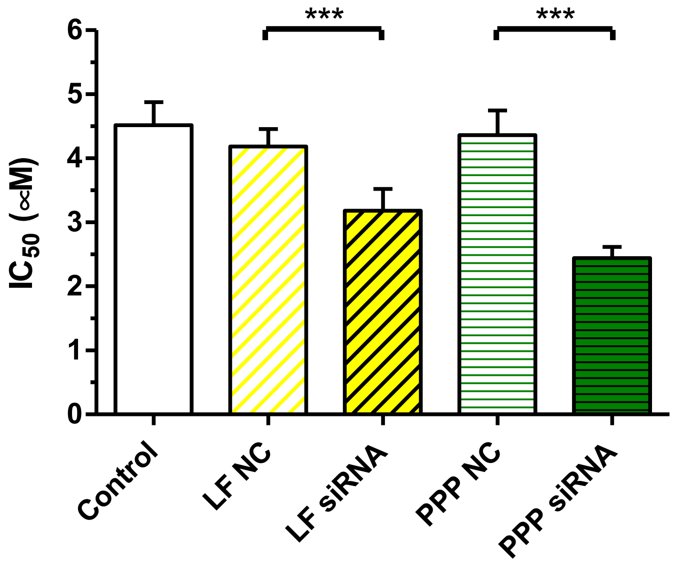

Figure S3. Statistic evaluation of IC_{50} values from colony survival assays in A549 cells following transfection with Lipofectamine 2000 (LF) or micelleplexes (PPP). Results are represented as mean ± SD.

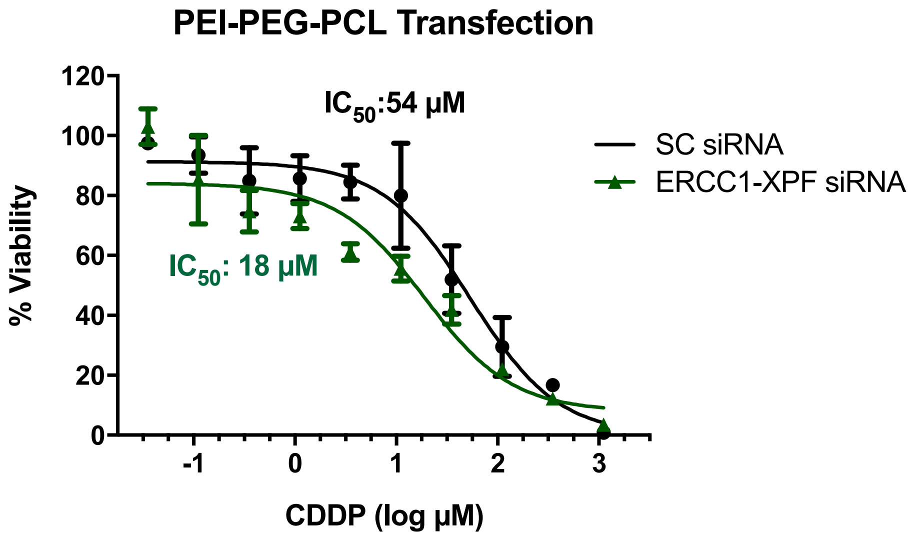

Figure S4. MTT assays in A549 cells following transfection with micelleplexes (PPP)containing negative control scrambled (SC) siRNA or siRNA against ERCC1 and XPF. Results are represented as mean ± SD.

© 2020 by the authors. Submitted for possible open access publication under the terms and conditions of the Creative Commons Attribution (CC BY) license (http://creativecommons.org/licenses/by/4.0/).
